# Carbon quantum dots as versatile nanomaterials for improving soil health and plant stress tolerance: a comprehensive review

**DOI:** 10.1007/s00425-025-04758-2

**Published:** 2025-07-09

**Authors:** Nida Andleeb, Saira Zafar, Zainab Rahim, Muhammad Mubashar Iqbal, Rattan Lal, Muhammad Ansar Farooq

**Affiliations:** 1 Institute of Environmental Sciences and Engineering, School of Civil and Environmental Engineering, National University of Sciences and Technology, Islamabad, 44000 Pakistan; 2 Institute of Soil and Environmental Sciences, Faculty of Agriculture, University of Agriculture, Faisalabad, 38000 Pakistan; 3 CFAES Rattan Lal Center for Carbon Management and Sequestration, School of Environment and Natural Resources, The Ohio State University, Columbus, OH 43210 USA

**Keywords:** CQDs, Nanomaterials, Soil health, Plant stress, Sustainable agriculture

## Abstract

**Main conclusion:**

This review summarizes the synthesis, purification/separation, and characterization techniques for carbon quantum dots (CQDs) and highlights their versatile applications as low-toxicity, multifunctional nanomaterials with particular focus on improving soil health, plant stress tolerance, and promoting sustainable agriculture.

**Abstract:**

Carbon quantum dots (CQDs) have recently emerged as highly versatile nanomaterials with a broad spectrum of applications. This review provides a comprehensive overview of various aspects of CQDs, including their synthesis via both top–down and bottom–up approaches, along with the critical separation and purification techniques required to fully harness their potential. This article highlights the multifaceted roles of both pristine and engineered CQDs in soil and plant systems, with a particular emphasis on soil biochemical processes and different stages of plant life cycle. A special attention has been given to the role of CQDs in enhancing plant tolerance to various biotic and abiotic stresses, such as nutritional imbalance, salinity, drought, heat, and heavy metal stress, with studies showing an improvement in stress tolerance, and considerable increase in seed germination rates when seeds are primed with CQDs. We have also discussed the crucial role of CQDs-mediated gene delivery in plants along with their influence on various soil characteristics. Moreover, the application of CQDs in the detection of agrochemical residues has been discussed with detection sensitivity reported as low as 0.1 µg L^−1^ for certain pesticides along with their broader utility in environmental remediation, food safety, and therapeutic applications demonstrating their unique properties and multi-functionality for sustainable use. This review also addresses the emerging concerns surrounding the potential toxicity of CQDs, particularly in plants, the food chain, and human health. While most studies report low toxicity at concentration below 100 mg L^−1^, understanding these risks is important to ensure the safe deployment of CQDs in real-world scenarios. In short, advanced research knowledge is provided as a basis for CQDs future development research toward efficient non-toxic delivery system for sustainable agriculture and other applications.

**Highlights:**

- Comprehensive exploration of synthesis methodologies for CQDs.
- The multifaceted role of CQDs in improving soil health and plant stress tolerance is discussed.
- Explores the nutritional significance of CQDs-coated fertilizers and their potential to revolutionize agricultural practices.
- Highlights the diverse application of CQDs in environmental remediation and biomedical field, emphasizing their cross disciplinary impact.

**Graphical abstract:**

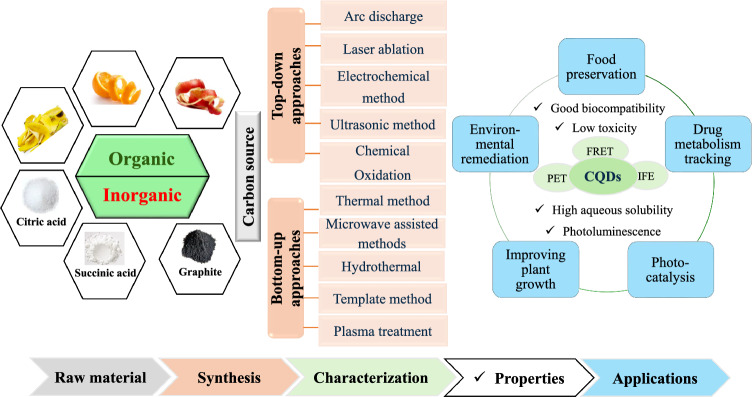

## Introduction

In recent years, nanomaterials have gained considerable attention owing to their usefulness in various fields (Dimkpa and Bindrban 2017; Omran 2020; Salem et al. 2022). They belong to the class of materials which have unique biological, chemical, and physical characteristics relative to their bulk forms and measure between 1 and 100 nm in size (Salem et al. 2022). The use of nanomaterials has revolutionized various industries by innovating areas, such as drug delivery, energy storage, material synthesis, etc., due to their small size, high surface-to-volume ratio, and special optical qualities (Ioannou et al. 2020). Among nanomaterials, carbon-based nanomaterials (CNMs) have gained much popularity due to their unique physico-chemical and optical characteristics (Tavan et al. 2025). Examples of CNMs include carbon nanotubes (CNTs), carbon quantum dots (CQDs), graphene quantum dots (GQDs), and polymer dots (PDs), etc. (Rao et al. 2018; Younes et al. 2019; Díez-Pascual 2024; Magesh et al. 2022; Jayaprakash et al. 2024). The existence of various types of CNMs reflects the possibilities and ease with which the size and properties of these versatile materials can be modified and tailored for specific applications. Among CNMs, CNTs represent cylindrical tubes of graphene, with low water solubility and biocompatibility but excellent electrical conductivity—limiting their suitability for reinforcement materials as well as nanocarriers (Mittal et al. 2020) (Table 1). Conversely, other types of CNMs, viz*.,* CQDs, GQDs, and PDs, slightly differ in their structural properties but usually have moderate-to-high-water solubility as well as biocompatibility (Li et al. 2010) (Table 1). In addition to that, due to their good optical properties (photoluminescence, electrical conductivity, tunable emission, etc.), CNMs demonstrate a wide array of applications in biomedical, environmental remediations, and agricultural sustainability (Li et al. 2010; Mittal et al. 2020).

**Table 1:** Types of carbon-based nanomaterials and their characteristics including different fields of application

Sr no	CNM type	Structure	Bio-compatibility	Toxicity	Water solubility	Optical properties	Applications	References
1	CNTs	Cylindrical tubes of graphene	Moderate	Moderate to high	Insoluble	Excellent electrical conductivity	Nanocarriers, reinforcement materials	Rao et al. 2018; Díez-Pascual 2024; Jayaprakash et al. 2024
2	CQDs	Spherical, amorphous or crystalline core	High	Low	Water-soluble	Strong photoluminescence, tunable emission	Biosensing, bioimaging, agriculture	Díez-Pascual 2024; Li et al. 2010
3	GQDs	Graphene-based sheets < 10 nm	High	Low to moderate	Moderate	Photoluminescence, good charge transport	Imaging, drug delivery	Díez-Pascual 2024; Jayaprakash et al. 2024; Wang et al. 2019a, b
4	PDs	Polymer-based dots	High	Very low	High	Bright luminescence	Biomedical, environmental	Díez-Pascual 2024; Jayaprakash et al. 2024; Wang et al. 2019a, b

To this end, CQDs in their pristine as well as functionalized forms exhibit excellent aqueous solubility and fluorescence characteristics suitable for broad range of applications (Díez-Pascual 2024; Kumar et al. 2022; Jan et al. 2024; Dong et al. 2024). Recent advances in nanotechnology have enabled facile, low-cost synthesis of CQDs that are typically < 10 nm in size (Cui et al. 2021; Guan et al. 2023). When compared to other CNMs, these zero-dimensional nanomaterials possess several advantageous characteristics, such as high stability, strong photoluminescence, and excellent biocompatibility that render them suitable for use in biosensors, bioimaging, environmental monitoring, food packaging, safety, and quality monitoring (Cui et al. 2021; Guan et al. 2023; Harish et al. 2022; Rasheed et al. 2023). Based on high-water solubility coupled with low cytotoxicity, the potential use of CQDs for sustainable crop production is an emerging area of research (Li et al. 2010; Boruah et al. 2022).

Pristine and engineered CQDs have been reported to impart multiple *ex-planta* and *in-planta* benefits that increase plant growth and productivity under normal as well as stress conditions (Nowack and Bucheli 2007; Riding et al. 2012; Li et al. 2010). CQDs application is reported to improve soil health by modulating soil microbial activities, rhizospheric pH, enzymatic activities, as well as soil carbon and nutrient dynamics (Vijeata et al. 2022; Sadeghi et al. 2023). Seed priming with CQDs has been reported to increase seed germination and early plant growth. Their hydrophilicity enables absorption of water and germination of seeds, having a favorable influence during early stages of plant life (An et al. 2020; Ashoknarayanan et al. 2021). The nutritional significance of CQDs-coated fertilizers as efficient carriers of nutrients and tailored nutrient supply is an important area of research (Elemike et al. 2019; Li et al. 2020a, b). Plants absorb CQDs from soil as well as from leaves via foliar application. Plant uptake and transport of CQDs is mediated through mechanisms, such as carrier proteins, ion channels, plasmodesmata, and endocytosis (Lin et al. 2009; Chen et al. 2010). Once taken up by plants, CQDs impart numerous benefits including enhanced tolerance against multiple biotic and abiotic stresses (Li et al. 2010; Younes et al. 2019). These positive effects could be ascribed to the impact of CQDs in driving photosynthetic processes and in collecting solar energy (Maholiya et al. 2023). CQDs-induced antioxidative and antimicrobial properties help reduce the adverse impact of environmental stressors, which in turn improve crop health and yield. Other known innovations include the role of CQDs as nanocarriers for gene delivery in plants, agrochemicals residues detection, use in mitochondrial nano-pesticides and nano-emulsions, which are environmentally friendly alternatives to traditional methods (Li et al. 2016; Ndolo et al. 2019; Mittal et al. 2020).

The role of CQDs as versatile nanomaterials in plant science depends on their unique surface properties and other physico-chemical characteristics. Therefore, the choice of synthesis methods determining shape, size, optical properties, and surface chemistry of CQDs should be carefully made. Eco-friendly synthesis methods should be employed for maximum agronomic and environmental benefits. With this aim, this review comprehensively covers the latest literature on the multifunctional roles of CQDs in sustainable agriculture. Besides the synthesis methods, sources, and the applications in plant stress management, there is an expanded coverage of topics not covered in previously published reviews on CQDs. These encompass the interactions of CQDs with the soil environment, focusing on their impact on soil health. In addition, the article discusses the mechanisms of CQDs-induced plant stress management, their role as nanocarriers in gene delivery, and sensing agrochemical residues and contributes to the development of precision farming. Furthermore, an overview of emerging applications of CQDs in food technology, and biomedical and environmental remediation is given. A section has been dedicated to toxicity and environmental impacts of CQDs as an attempt to guide future research toward the safe and sustainable application of these nanomaterials in plant stress management and beyond.

## Synthesis, separation, purification, and characterization techniques for CQDs

### Synthesis techniques

Since the invention of CQDs, their physico-chemical attributes, such as surface area, optical absorbance, photoluminescence, electroluminescence, and the distribution of surface functional groups, have undergone modification using various synthetic techniques (De Medeiros et al. 2019). The choice of synthetic techniques is determined by specific applications in food industry, biomedical, agriculture, and environmental remediations (Guan et al. 2023). It is worth noting that the efficacy and usefulness of CQDs is greatly impacted by the post-synthetic treatments, especially the separation and purification steps, which are outlined in detail in later sections.

**Fig. 1:**
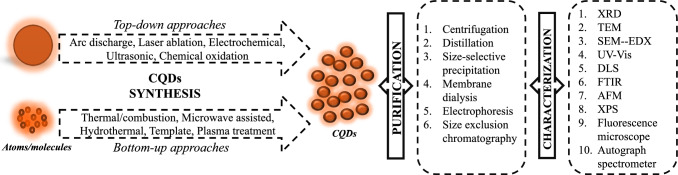
An overview of CQDs processing techniques, viz*.,* synthesis via top–down and bottom–up approaches, purification, and characterization

CQDs can be synthesized via two principal approaches: the top–down and bottom–up approaches (Fig. 1). Machines employing physical, chemical, and/or electrochemical processes can be instrumental (Magesh et al. 2022). In the top–down method, techniques like arc discharge, laser ablation, electrochemical exfoliation, and ultrasonic and chemical oxidation methods are employed in the disintegration of large carbonaceous materials to nanoscale powders. While in the bottom–up method, small organic molecules or even individual atoms are bonded chemically to synthesize carbon nanostructures through pyrolysis and hydrothermal or solvothermal processes, or microwave-assisted, template-based, and plasma treatment techniques (Fig. 1). Chemical aerobic or thermal energy inputs usually power this assembly (Arole et al. 2014). All of these strategies have unique limitations and benefits, such as cost, scale, environmental issues, and manipulation of CQDs characteristics. A summary comparison of top–down and bottom–up synthesis techniques for CQDs with distinct quantum yield, and size range along with advantages and disadvantages of each technique is provided in Table 2.

**Table 2:** Top–down and bottom–up synthesis techniques for CQDs with each method having its own advantages and disadvantages

Sr. no	Synthetic techniques	Size range (nm)	Quantum yield (%)	Advantages	Disadvantages	References
1	Arc discharge	1–5	16	Most attainable method	Harsh conditions possess low quantum yield and composite method	Biazar et al. 2018; Chao-Mujica et al. 2021; Dey et al. 2014
2	Laser ablation	5	4–10	Effortlessness, effective technique, tunable surface states	Large amounts of carbon matter is required, poor control over sizes, low quantum yield	Koshizaki et al. 2011
3	Electrochemical	6–8	2.8–8.9	Stable method, extent of CDs can be managed by changing current density, water-soluble CDs can also be prepared	Complex method	Anwar et al. 2019
4	Ultrasonic method	1–6	60–65	Water-soluble, radioactively stable, weakly toxic and photostable,	Requires high energy, pressure and temperature	Huang et al. 2018; Kumar et al. 2020; Yang et al. 2016
5	Chemical Oxidation	3.2	8.6	Enhance water dispersibility, strong fluorescence, high stability and high purity	Non-uniform size distribution, use of aggressive oxidizing agents	Feng et al. 2019; Iannazzo et al. 2018; Malavika et al. 2022
6	Thermal/combustion technique	2–6	0.1–3	Easy and straightforward method, have fluorescence property, appropriate method for particles (on milligram scale)	Low quantum yield	Arole et al. 2014; Bera and Aruna 2017; Thoda et al. 2018
7	Microwave-assisted	4–5	4–6.1	Simple and convenient method, inexpensive and eco-friendly method	Poor control over sizes	Medeiros et al. 2019; Romero et al. 2021
8	Hydrothermal and aqueous-based	10	Up to 90	Highly water dispersible carbon dots can be prepared, inexpensive, non-toxic	Poor control over sizes	Shen et al. 2018; de Yro et al. 2019
9	Template-based	2–5	22	CDs have biocompatibility and colloidal stability	Time-consuming and expensive method have limited quantum yield	Du et al. 2021; Hong et al. 2019; Zhang et al. 2015
10	Plasma treatment	1–2	5	Monodispersed in size, good water solubility, CQDs will be rich in oxygen content, Fluorescent	Complex and high-cost restrictions	Dager et al. 2020; Ma et al. 2019

## Top–down approaches

### Arc discharge method

The process which begins with an arc discharge or electrical breakdown of gas, leading to the formation of plasma, is subdivided into steps. A chamber with two electrodes set horizontally or vertically is built for this technique. The anode is filled with a powdered carbon precursor and a metal catalyst, while the cathode is a pure graphite rod. The chamber may be filled with gas or liquid. For starting the arc, electrodes are first touched and then pulled away to a fixed distance of 1–2 mm (Chao-Mujica et al. 2021). To prevent and sustain continual, non-oscillating discharge, the electrodes are dynamically held at this distance by automated closed loop systems. This leads to plasma being produced at a remarkably high temperature (around 4000–6000 K). The energy temporarily stored in the arc is used to vaporize the carbon precursor contained within the anode. The gas phase carbon, which is supercooled by now, moves toward the temperature of the colder electrode, solidifying at that electrode’s surface resulting in the created carbon being condensed.

After a few minutes of applying the arc, the discharge process is stopped, and the soot containing the NPs (with CQDs included) is scraped off the chamber walls (Arora and Sharma 2014). Due to the different sizes of carbon particles produced during the discharge process, the CQDs produced by this method are of a wide particle-size distribution. Quasi-spherical CQDs suffer from too high average particle sizes, which greatly reduce the specific surface area, thus may lessen the active reaction sites during the electrocatalytic process (Rasal et al. 2021).

### Laser ablation method

Laser ablation (LA) is a new method which shows promise in preparing NMs and is particularly suitable for CQDs because of the rapid rate of processing (Xu et al. 2019). In the process of LA, a solid target is subjected to a high-powered laser, such that it generates zones of localized high pressure and temperature. This enables pulverization of the solid target material into nano- or micrometer-sized particulates, and thermodynamic residues are not an issue. Plasma is generated in the region, which cools down and crystallizes to yield pure nanoparticles (Cui et al. 2021). This is a very efficient method for producing CQDs that can have narrow size distribution (as low as 5 nm), are highly water soluble, and have favorable fluorescent properties such as quantum yield approaching 40%. This method does have some drawbacks, such as the notorious need for large volumes of precursor bulk material, overly complex processes, and exorbitantly high prices. Although, this method does show promise compared to others (Gonçalves et al. 2010; Goodwin et al. 1997; Singh et al. 2012). Cui et al. (2021) employed this technique for synthesized homogeneous CQDs with ultrafast dual-beam pulsed LA for bioimaging of plant and animal cells.

### Electrochemical method

The electrochemical technique entails carefully regulating the reduction or oxidation of electricity to carbon containing precursors to produce carbon-based materials on the nanometer scale. For this purpose, an electrochemical cell is made by taking a certain carbon precursor as the cathode or anode and placing it into a bath of non-aqueous or aqueous solvent based on the intended qualities of the resulting CQDs (Wang 2022). The reason why electrolyte formulation is important is that it determines the properties of the synthesized CQDs. Their properties are given by the control of the crucial parameters in the electrochemical synthesis which are temperature, reaction time, current density, and voltage. These parameters can control the morphology, surface properties, and the particle-size distribution of the CQDs produced (Kalita et al. 2020). This method is very useful, since it is simple and can be carried out at room temperature and pressure.

It is reported that some particles of the synthesized CQDs can have a photoluminescence yield between 2.8 and 8.9% (Anwar et al. 2019). Blue-emitting CQDs with an average particle size of 2.4 nm were produced by electrochemical carbonization of urea and sodium citrate in deionized water (Hou et al. 2015). It has properties that make it a highly sensitive detector of mercury ion in wastewater and plants. Although this procedure is relatively commonplace to produce high-performance electrocatalysts, the application of CQDs as electrocatalyst is rather uncommon (Thakur and Kumar 2022).

### Ultrasonic method

Ultrasonication is an efficient method for synthesizing carbon quantum dots (CQDs) due to its low cost and speed as well as relatively low ecological impact (Li et al. 2011; Huang et al. 2018). This method starts with the opening and closing of a bubble when a liquid is subjected to a high and low ultrasonic intensity, which starts low and high oscillating sound waves (Li et al. 2011). After rolling and collapsing these gases or tiny bubbles strengthen by all cavitation leading to creating an environment of high temperature as well as high pressure and sudden breakdown of dormant carbon ancestors along with extensive raising of CQDs suspended growth environment. Managing several reaction parameters, including duration of reaction, ratio of carbon source, solvent, pumping power, and other ultrasound sources, respectively, permits accurate control on CQDs properties. Numerous researchers have shown food waste, carbon fibers, multiwall carbon nanotube, and even graphitic materials to be very effective for CQDs synthesis (Li et al. 2011; Wang et al. 2019; Yang et al. 2016). The waste-soluble CQDs turn out to be radioactively stable and luminescent while being weakly toxic and photostable, were produced when food waste was sonicated in ethanol (Baker and Baker 2010). In addition, nitrogen and sulfur can be used to easily fabricate heteroatom-doped CQDs, which alter the characteristics of these due structured CQDs and their optical and electronic properties. Most of the luminescent doped CQDs are blue and behave in a particulate like manner with a diameter of about 1 to 6 nm (Huang et al. 2018).

### Chemical oxidation method

This method is important for CQDs synthesis due to its effectiveness in converting bulk carbon to nanoparticles (Rasal et al. 2021). Using this approach to produce CQDs is favorable because of the greater simplicity, better efficiency, and higher level of feasibility accompanying the method. Beyond just reducing the size of carbon materials, this process adds hydrophilic functional groups such as –OH and –COOH, which enhances the dispersibility of CQDs in biological and aqueous environments. Examples of strong oxidizing reagents which can oxidize carbonaceous constituents of Chinese ink and coke include sodium chlorate, nitric acid, and sulfuric acid (Dong et al. 2010; Yang et al. 2014). Hydrothermal reactions with oxidized carbon nanoparticles and precursors with sodium hydrosulfide dimethyl formamide and sodium selenide enable the introduction of heteroatoms such as selenium, sulfur, and nitrogen. The photoluminescence and quantum yield of CQDs are significantly improved when heteroatoms are incorporated into the structures of CQDs (Yan et al. 2014). These CQDs obtained from acidic oxidation are reported to be used ionically for electrocatalysis and drug delivery, in addition to being utilized in electrochemical sensing and cell imaging (Feng and Zhang 2019; Iannazzo et al. 2018). Nevertheless, this approach is not without its drawbacks, because it entails the use of aggressive oxidizing agents which need to be closely monitored and requires a lot of processing to yield high purity CQDs.

## Bottom–up approaches

### Thermal/combustion method

This burns the precursor material to combust liquids into gases which can be functionalized with different groups and conjugated (Arole and Munde 2014). Surface functionalized carbon nanoparticles are produced by the thermal carbonization of a myriad of ammonium citrate salts. In particular, the carbonization of octadecyl ammonium citrate salt results in the formation of organophilic CQDs, whereas the use of 2-(2-aminoethoxy)-ethanol salt yields hydrophilic CQDs. During the process, the thermal dehydration of the ammonium carboxylate fragment results in the bond formation of –NHCO– between the organic moiety and carbon core (Tian et al. 2020). In addition, it has been reported that the photoluminescence features of the end products are greatly dependent on the reaction temperature. Following this, these CQDs need to be dialysed to remove any residual contaminants. Alternatively, it is achievable to produce monodispersed CQDs with an average diameter of less than 10 nm by varying the parameters of the experiments. It offers high surface area electrostatic active materials in the form of nanoparticles with abundant functional groups which enhance their performance in electrochemical processes (Bera and Aruna 2017; Thoda et al. 2018). Besides being elementary and easily adaptable, this technique is also precise, low-cost, and non-polluting, making it much more attractive than the current available methods for producing large quantities of CQDs (Arole and Munde 2014).

### Microwave-assisted method

For the microwave-assisted synthesis of carbohydrate quantum dots (CQDs), the precursor materials, which typically include saccharides, are first dissolved in water and then microwaved. This method permits the formation of various morphologies of CQDs, such as hydrophobic, hydrophilic, and amphiphilic. This is a simple, fast, and low-cost CQDs fabrication technique which can be performed with little-to-no care to waste management to obtain oxygen-doped CQDs that serve as metal-binding sites for carbon-based electrocatalysts (Medeiros et al. 2019; Zhao et al. 2019). Tan et al. (2021) reported the extraction of CQDs from empty fruit bunch (EFB) biochar using a greener acid-free microwave technique. Enhancing the application of CQDs on rice (C3) and corn (C4) leaves were studied using their ability to assimilate carbon dioxide (CO_2_) and then measuring stomatal conductance. They concluded that for the artificially grown crops, the incorporation of CQDs in their cultivation significantly enhances their rate of photosynthesis, showcasing the potential role of CQDs in agriculture.

### Hydrothermal and aqueous-based method

According to Singh et al. (2018), the most employed techniques for producing CQDs include polymerization, dehydration, passivation, and carbonization. These techniques generally begin by mixing small organic molecules and polymers with either water or an organic solvent to form a precursor solution that is subsequently loaded into a stainless-steel autoclave Teflon case. Organic compounds and/or polymers offer thermally decomposed at elevated temperatures to generate CQDs with a core seed of carbon which have sizes below 10 nm (Shen et al. 2018; de-Yro et al. 2019). Han et al. (2020) synthesized CQDs using hydrothermal processes and analyzed their use as probes for chlorogenic acid in coffee and honeysuckle. The simplicity with which CQDs can be synthesized and heteroatom doped makes this method one that will accelerate the development of electrocatalysts with designed structural and compositional features.

### Template method

Using this procedure, a surrogate in the form of polymer, surfactant, or even a biological molecule can be used to control the dimensions, shape, and surface features of CQDs. A constructed mold serves as a matrix which guides the CQDs to take the desired shape. Generally, the CQDs form through the carbonization of precursor molecules, organic or inorganic compounds, within a template. The template allows both templates assisted growth and nucleation which results in well-defined CQDs with specified surface chemistry, optical characteristics, and dimensions. Assisted synthesis is one parameter which along with the type of precursors employed and the template used, can be modified to change CQDs characteristics thus improving their functionality in drug delivery, optoelectronics, and bioimaging (Hong et al. 2019; Du et al. 2021; Rasal et al. 2021).

### Plasma treatment

With plasma technology, Jiang et al. (2010) found a single-step approach to producing monodisperse CQDs which was previously thought to be impossible. Surface functionalization and fabrication are accomplished simultaneously in this approach. Free radicals were generated onto the surface of CQDs using a submerged arc plasma reactor, which is easier to be functionalized with EDTA in a benzene solution.

## Eco-friendly synthesis approaches

Conventional synthesis techniques, i.e., chemical oxidation, microwave-assisted, or hydrothermal techniques, are common ways to synthesize nanomaterials, but it often leads to the production of dangerous byproducts, consumes a lot of energy, and makes the process less eco-friendly. Environmentally compatible synthesis techniques, mainly those based on green chemistry, have sustainable options to use biological materials and moderate reaction conditions (Manikandan and Lee 2022). Green synthetic techniques use substances from plants, bacteria, fungi, and algae to produce CQDs. They are gaining popularity, because they are cost-effective, easy to use, could have large-scale production and friendly to the environment (Manikandan and Lee 2022). Extracts from different plants have a wide variety of phytochemicals, such as flavonoids, phenolics, and terpenoids which support both the removal of metal ions and improve the nanoparticle properties, making them more stable and functional (Malavika et al. 2022).

Similarly, some microbes like *Bacillus subtilis*, *Aspergillus niger*, and *Chlorella vulgaris* create nanoparticles inside their cells or outside, making the synthesis process controlled and allowing us to manage the shape and size of the nanoparticles (Koul et al. 2021). A study reported the green synthesis of N-doped CQDs from *Moringa oleifera* roots as a precursor. Their application to sorghum seedlings resulted in extremely low toxicity of N-CQDs and they also facilitated pathogen detection, i.e., sulcotrione. The study also concluded that CQDs have a great potential to be used as green pesticides (Wang et al. 2021a, b).

## Separation, purification, and characterization techniques of CQDs

### Separation and purification techniques

CQDs are often found in emulsions rather than monodispersed or fully purified. CQDs often appear in the form of aggregates or coagulates, so their surface properties and functionality can be maximized only if effective purification and separation methods are observed. Techniques of separation or purification can vary based on the synthetic method used and the properties of the CQD’s surface. Different methods of centrifugation, distillation, size-selective precipitation filtration, membrane dialysis or other electrophoresis, and size-exclusion chromatography (SEC) are being utilized for the CQDs’ purification and separation (Alamo-Nole et al. 2012; Carrillo-Carri on et al. 2011; Shen et al. 2013).

Sun et al. (2023) used membrane dialysis on CQDs doped with nickel (Ni) that were synthesized using electrochemical techniques. The Ni-CQD powder was freeze dried and the electrolyte solution was dialyzed in DI water using a dialysis bag (3500 Da). Zhou (2021) described that differential centrifugation makes it possible to achieve size-dependent tenability for property specific perovskite QD-based optoelectronic devices which makes the process novel. Vibhute et al. (2022) used centrifugation to purify the CQDs synthesized from 1,5-diaminonaphtaline (DAN) and citric acid by the hydrothermal technique, so the CQDs were monodispersed and purified.

### Characterization techniques

CQDs formed from different precursors can be found in amorphous and graphitic structures. Sizes of CQDs may be achieved using different nanocomposites. Various methods are used to characterize CQDs which include X-ray diffraction (XRD), transmission electron microscope (TEM), UV–Vis, fluorescence, dynamic light scattering (DLS), Fourier Transform Infrared (FTIR) autograph spectrometer, atomic force microscope (AFM), and X-ray photoelectron spectroscopy (XPS) (John et al. 2022).

The average number (NAD) and weight (WAD) distributions of CQDs are calculated from AFM and TEM while their stoichiometric composition is substantiated by XPS and energy dispersive X-ray spectroscopy (EDX). XRD reveals whether the CQDs are crystalline or amorphous. FTIR methods confirm CQDs with such functional groups as C–O–C, COOH, C–OH, C–H, and C–C. Fluorescence and scan of ultraviolet–visible (UV–vis) are used for studying optical properties of CQDs (Choppadandi et al. 2021).

## Structural features and potential properties of CQDs

The way CQDs make possible the retrieval of stressed plants is simply unique due to their structural characteristics. These include size and shape, adjustable fluorescence emission with enhanced photo stability, increased light absorption, and high-water solubility. Furthermore, CQDs are quite favorable in agriculture owing to their possibility of efficient electron transfer as well as up-conversion (Li et al. 2019; Li et al. 2023a, b). One of the most prominent features of CQDs is their nanoscale dimensions, which give them remarkable physical and chemical properties with numerous applications. Several reports have underscored the importance of CQDs size on their mobility within the plant systems, and, consequently, the translocation of nutrients both in normal and stress conditions. These unique characteristic CQDs enable them to enter plant cells through open pores and cavities between cells. For instance, 4 nm CQDs were reported to swiftly move through mung bean seeds and eventually spread into the root, stem, and leaf. Unlike the former, other CQDs with 5 nm average sizes are known to be stuck in the leaf stomata and cell walls of wheat plants (Gong and Dong 2021).

Another important feature of the structure of CQDs is the binding positions of surface functional groups, such as -COOH, -OH, and -NH_2_, which are known to be important in relation to plants. The antioxidant activity of these functional groups is further increased along with their fluorescence and electron transfer activity. Indeed, tolerance to a wide range of heavy metal stress (et al. Lu et al. 2025; Falak et al. 2025; Chai et al. 2024) is provided in addition to improving photosynthetic performance. With the help of such functional groups as -NH_2_ and -COOH, the CQDs possess remarkable ability to scavenge ROS, which relieve stress on the plants (Singh et al. 2024). Furthermore, due to their remarkable fluorescence capable of monitoring plant metabolites and detecting agrochemicals, CQDs possess multifunctional potential in agriculture.

## Synthesis sources

The synthesis of nanomaterials through carbon-rich substances such as renewable biomass is possible. The scope of available precursors permits CQD properties to be modified effectively and sustainably. Because of their renewability, the biomass byproducts have attracted considerable interest and make such materials advantageous for the industrial-scale production of CQDs.

### Natural or biomass products

Rather recent research has sought to advance methods for synthesizing carbon precursors that are cost-effective and environmentally benign due to the prospective harm on flora, humans, and the soil ecosystem from the toxic chemicals used in manufacturing CQDs. Non-toxic biomass-derived products as carbon precursors are ideal, since they are rich in oxygen, heteroatoms, and carbon. These sources are relatively inexpensive, easier to procure, and environmentally friendly compared to traditional alternatives, making them commercially viable (Gao et al. 2017; Kong et al. 2024). Using waste biomass for the fabrication of CQDs contributes to waste management and the green economy and is also associated with enhanced photoluminescent properties (Liu et al. 2019).

Biomass-sourced CQDs have demonstrated considerable potential in different studies. For example, Jagannathan et al. (2021) synthesized white-emissive CQDs from burning corn seeds. In Shahba et al. (2020), CQDs derived from pinecones using ball-milling and hydrothermal techniques exhibited higher photocatalytic activities, as well as higher adsorption capacity for Cd ions (Li et al. 2018a, b). Liu et al. (2012) produced nitrogen-doped photoluminescent polymer nanodots from grass and noted that the fluorescence intensity and quantum yield increased as the reaction temperature increased. Raikwar (2022) illustrated the bioimaging of plant cells and the manufacturing of nanocomposites using aloe-vera biomass thermal carbonized CQDs. Also, Nguyen et al. (2022) produced blue, fluorescent CQDs from banana peels using a new one-pot hydrothermal approach, while Wang et al. (2020) produced CQDs from tangerine peels and ginkgo biloba leaves via hydrothermal treatment with a base. Finally, Wang et al. (2016) prepared wool-derived CQDs through microwave pyrolysis, yielding very monodisperse and stable nanoparticles. Boruah et al. (2020) produced CQDs from taro scalp, garlic skin, and sugarcane bagasse via an ultrasound-assisted wet chemical oxidation process, while Xu et al. (2021a, b) produced CQDs from an oak cup. Conducting biomass-sourced CQDs research enables progress toward sustainability, because, in most instances, their precursors stem from biological origin, primarily plants and animals.

### Other carbon sources

Zhou et al. (2007) pioneered the synthesis of CQDs via electrochemical method in a carbon vapor deposition system with multiwall carbon nanotubes (MWCNTs) acting as the working electrodes. The CQDs produced were noted to fluoresce blue. Liang et al. (2023) synthesized CQDs from plastic waste to be utilized as nano-inhibitors that enhanced germination of pea seeds and increased the amount of water in the roots. Huo et al. (2015) used sodium hydroxide and acetone as precursors and synthesized CQDs that resulted in dark brown solid products. Ren et al. (2019) work on nitrogen-doped CQDs showcased the use of the pulsed laser ablation (LA) technique with formulated bellflower wood which achieved a dual-wavelength photoluminescence emission with quantum yield of 32.4%. Small organic molecules are often used as precursors in the process for synthesizing CQDs. Zheng et al. (2015) developed a simple technology to produce luminescent CQDs from small organic molecules containing vitamin moiety and a benzene ring. Similarly, Yu et al. (2015) used unfocused laser irradiation for the synthesis of CQDs from toluene. In sharp comparison, Zhai et al. (2012) derived polyaspartic acid and glucose microwave-assisted carbonization technique CQDs. Table 3 summarizes a comparison of different precursors of CQDs synthesis, i.e., biomass-derived, synthetic, industrial waste in terms of their cost, environmental impact, and quality of synthesized CQDs.

**Table 3:** A comparison of different precursors used for CQDs synthesis, i.e., biomass-derived, synthetic, and industrial waste

Sr no	Precursor type	Cost of synthesis	Environmental impacts	Quality of CQDs	References
1	Biomass derived	Low	Very low- (biodegradable and renewable)	Moderate to high (depending on source)	Wang et al. 2020; Nayak et al. 2025
2	Synthetic	High (pure chemicals are used)	Moderate to high (toxic solvents/byproducts)	High (controlled size & purity)	Cui et al. 2021
3	Industrial wastes	Very low	Low to moderate (needs pre-treatment)	Variable (depends on waste type)	Parveen et al. 2025

## Uptake, transport, and accumulation of CQDs in plants

**Fig. 2:**
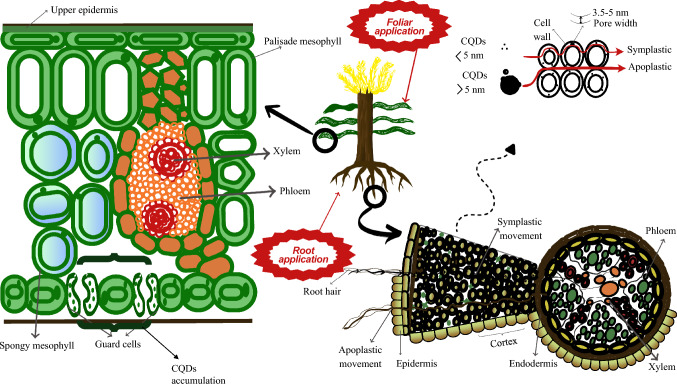
Schematic representation of the potential uptake and translocation pathways of CQDs in plants. CQDs can be introduced through foliar application or soil application. Uptake occurs via root hairs in the soil or through leaf surfaces, where CQDs can penetrate through the cuticle and stomatal pathways. Once inside the plant, CQDs are transported through apoplastic and symplastic pathways, reaching different tissues, including xylem and phloem, facilitating distribution all over the plant

CQDs usually enter plant systems through roots’ absorption or foliar application. Different types of microscopes can be used to trace CQDs in plants to understand their uptake, translocation, and accumulation in plants. Figure 2 illustrates that CQDs are absorbed by root hairs and then transported to vascular bundles, i.e., xylem and phloem of stem and leaves. The pathway through which CQDs will move inside plant body mainly depends on their size and surface functional properties. Plant cells have several pores in their cell wall and their pore width is usually between 3.5 and 5 nm (Carpita et al. 1979). CQDs with sizes less than 5 nm enter cells through these pores of cell wall via cell membrane while CQDs with sizes greater than 5 nm are unable to penetrate these pores and get adsorbed on the outside surfaces of cell walls. Root hairs in plants are responsible for the absorption of water and nutrients from the soil and this high specific surface area of root hair is responsible for CQDs absorption from soil to plants. This flow of water and nutrients facilitates the movement of CQDs in plants along with them. The CQDs (< 5 nm) adsorbed on the cell wall move through apoplastic pathway, i.e., movement through cell wall and intercellular spaces, while the CQDs (> 5 nm) inside the cell move through symplastic pathway, i.e., movement through cytoplasm via plasmodesmata (Khan et al. 2022). After passing through the whole cortex of roots, CQDs enter the vascular bundles and are transported to the aerial parts of plants, i.e., leaf veins and stomata. A study by Gong and Dong (2021) employed a wheat model for studying the transfer, transport, and accumulation of Ce-doped CQDs. The observation revealed that the dilated hair roots of wheat plants absorbed Ce-CQDs and they were subsequently translocated through the fibro vascular tissues of the stems and leaves via the vascular system. They suggested that this phenomenon may be due to two aspects. First, CQDs with small size accumulated on cell walls and roots, while large size CQDs failed to enter cells are transported to leaves through vascular bundles and get accumulated in their tissues. Second, the decomposition of some Ce-doped CQD during the upward translocation in plants reduce their concentration in aerial parts. Dong et al. (2022) confirmed that mung bean roots took up Ce-CQDs and they were subsequently translocated through the vascular system and apoplastic routes to the stems, intercellular spaces, cilia, stomata, and the veins of the leaves and aggregated in the vascular system (Fig. 2).

This information describes the great efforts that are being undertaken in understanding the processes involved in CQDs uptake, translocation, and accumulation inside the plants. The promising results obtained from this study could form the foundation for further research and development of CQDs tracking devices make accurate detection of the location of each signal possible within the plant systems. Moving forward in this space may open new ways to using CQDs in agriculture.

## CQDs impact on plant growth under normal and stress conditions

**Fig. 3:**
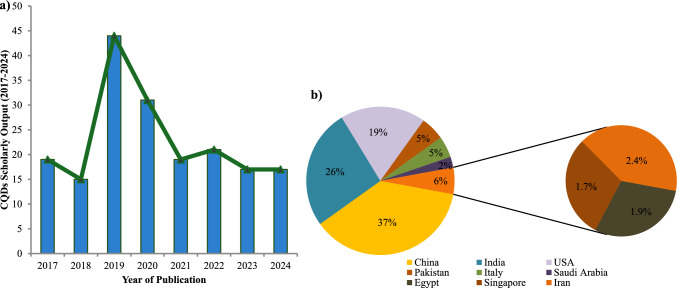
**a** A bar chart showing the trend in scholarly output on the applications of CQDs in agriculture, from 2017 to 2024. **b** The pie chart illustrates the geographical distribution of contributing countries to the scholarly output, highlighting global interest and involvement in CQDs-based agricultural studies

Literature surveys for scholarly publications on CQDs (2017–2024) indicate growing global interest in their application in agriculture due to their multifunctional roles (Fig. 3). Several studies have been conducted in various regions across the globe—investigating the versatile role of CQDs for enhanced seed germination through seed priming, improved nutrient use efficiency of CQDs-coated fertilizers, implications for soil health improvement, as well as their potential to improve plant resilience against (a)biotic stresses (Tables 4 and 5).

**Table 4:** Impact of seed priming with CQDs and doped CQDs and their effects on the overall plant’s life cycle particularly on seed germination

Sr. no	CQDs types	Source	Plant type	Type of stress	Treated plant stage	Priming concentrations	Effects on plants (increasing parameters)	Effects on plants (decreasing parameters)	References
1	Cerium (Ce)-doped CQDs (nanoceria)	Poly (acrylic acid)-coated Ce oxide nanoparticles (PNC)	Cotton (*Gossypium hirsutum* L.)	Salinity	Seeds	500 mg L^−1^ PNC in water (24 h)	Seedling root length (56%), root vitality (114%), fresh weight (41%), and dry weight (38%), modified root anatomical structure	ROS accumulation in seedling roots (46%)	An et al. 2020
2	Plastic-derived NPs (PDNs)	Polyethylene terephthalate (PET)	Peas (*Pisum sativum)*	–	Seeds	0.25–2 mg mL^−1^	Seed germination rate, shoot and root elongation, biomass accumulation, and root moisture levels	–	Liang et al. 2023
3	Zinc-doped CQDs	Zinc-oxide (ZnO)	Lupine plants (*Lupinus termis*), Black gram (*Vigna mungo)*	Salinity	Seeds	20, 40, 60 mg L^−1^	Photosynthetic pigments, adjusted osmoregulation	Lowered the contents of MDA	Latef et al. 2017
4	Putrescine-functionalized- CQDs (Put-CQDs)	Putrescine and citric acid	Grapevines (*Vitis vinifera)*	Salinity	Seeds	5, 10 mg L^−1^	Fresh and dry weight, enhanced ionic homeostasis, increased chlorophyll, and carotenoid concentrations, antioxidant enzymatic activities	Reduced cellular damage (lowering the concentrations of H_2_O_2_ and MDA), neutralized proline and total phenolic compounds	Gohari et al. 2021a, b
5	Graphene-doped CQDs	Graphene	Tomato	-	Seeds	10, 100, 250, 500, and 1000 mg L^−1^	Concentration of photosynthetic pigments (chlorophylls and carotenoids), enhanced enzyme activity	Reduced H_2_O_2_ concentrations	López-Vargas et al. 2020
6	Nitrogen-doped CQDs (N-CQDs)	Melamine and ethylenediaminetetraacetic acid	Tomato and corn seedlings	Biotic stresses (e.g., diseases)	Seeds	Tomato (10 mg L^−1^) Corn (50 mg L^−1^)	Enhanced ROS scavenging, exhibiting higher oxidative stress alleviation, activation of salicylic acid and jasmonic acid dependent systematic acquired resistance	Reduced ROS levels	Luo et al. 2021; Wang et al. 2021a

### Priming effects on seed germination

Seed priming with either pure CQDs or doped CQDs presents an innovative approach to enhancing early plant growth. This technique is highly effective in overcoming seed dormancy, promoting synchronized germination, and improving overall seedling health. Table 4 summarizes various plant species subjected to seed priming and its effects throughout their life cycle. Enhancement in plant morphological characteristics following CQDs treatment, highlighting their role as effective growth stimulators. Further to germination, these advantages of CQDs priming also include improved growth and developmental processes of the plant, increased tolerance toward different environmental stresses, early flowering, and eventually raised plant growth, development and productivity (Table 4).

### Nutritional significance of CQDs-coated fertilizers

Over the past few years, the application of CQDs in coating fertilizers has seen considerable growth as a method to enhance plant absorption of nutrients (Khan et al. 2022). CQD-coated fertilizers are superior to the modern and even contemporary polymer-type fertilizers because of their high surface area-to-volume ratio (Elemike et al. 2019). Compared to the traditional mixtures, these fertilizers allow a decrease with increased efficiency in the use of chemical inputs, improve stress resistance, and change the ecological impact for the better (Dimkpa and Bindraban 2017). Moreover, CQDs coated with fertilizers slow the releasing of nutrients to the plants to synchronically match with plant growth phases, ensuring adequate nutrition absorption and plant growth while limiting nutrient losses from poor soil interaction. Li et al. (2023b) observed that the application of selenium-doped CQDs (Se-CQDs) coated fertilizers resulted in indirect absorption by the plants enhancing the biomass yield, mineral constituents, and the quality of fruits. Likewise, Tan et al. (2023) synthesized water-soluble nano-fertilizers in the form of nitrogen-doped CQDs and greatly enhanced the chlorophyll contents, light energy conversion efficiency, Rubisco activity, and electronic transmission activity. Despite these positive outcomes, there still remains a gap to properly understanding the nutritional value of these CQD-coated fertilizers which need to be further investigated.

## Impact of CQDs on soil properties

Application of nanotechnology particularly CQDs into soil ecosystem is a growing area of research and has the immense potential to affect soil nutrient availability, microbial populations, and pollutant dynamics. Various soil aspects, such as soil pH, organic and inorganic compounds, and soil microbes such as bacteria, and fungi (e.g., arbuscular mycorrhizae), play a crucial role in modulating the behavior of nanoscale micronutrients and they can also affect CQDs by influencing their dissolution, aggregation, and surface properties (e.g., charge and coatings) potentially leading toward altered soil and plant outcomes. Therefore, evaluating the impacts of CQDs on soil environment is critical for determining CQDs role in sustainable agriculture practices. Next, we will look at how CQDs alter soil microbiota, pH/nutrient availability, and enzymatic activities.

### Influence of CQDs on microbial activity in the rhizosphere

Soil, as an open biogeochemical system, is a key recipient of nanomaterials and microbial activity is a critical indicator of soil health, reflecting the soil's response to environmental changes (Nowack and Bucheli 2007; Riding et al. 2012). CQDs owing to their nanoscale size can easily interact with soil microorganisms and thereby influence their growth and activities. For instance, Yin et al. (2024) reported enhanced diversity and relative abundance of soil bacterial communities such as *Actinobacteria* phylum by the application of selenium-doped CQDs (Se-CQDs), this phylum *Actinobacteria* is considered beneficial in plant growth promoting substances. Such rhizosphere microorganisms and root exudates, both play important roles in maintaining plant health and increasing plant tolerance to stress. Examples include root exudates such as organic acids (lactic acid, citric acid, and formic acid) and carbohydrates (glucoside) (Song et al. 2012; Xu et al. 2021b). In another study, Yang et al. (2022) documented significant increase in the microbial population of *Conocybe* (122.6%), *Nitrospira* (126.2%), *Sphingomonas* (233.3%), and *Pseudomonas* (344.4%). Their study demonstrated that foliar application of carbon-based nanomaterials synthesized via a hydrothermal method using citric acid and ethylenediamine as carbon sources increased root exudates, including succinic acid and pyruvic acid (tenfold increase), and betaine (11.8-fold increase). This enrichment of root exudates stimulated microbial growth in the plant rhizosphere, subsequently increasing soil available P by 16.8% and N by 33.5%. Similarly, Ji et al. (2023) found that foliar application of carbon-based nanomaterials increased root secretion (e.g., auxins, organic acids, and amino acids) and attracted beneficial microorganisms, such as *Ascomycota*, *Actinobacteria*, *Glomeromycota,* and *Acidobacteria*, facilitating soil N activation.

### CQDs role in modifying soil pH, carbon contents, and nutrient availability

The introduction of CQDs into the soil could change some of its fundamental features, such as soil C retention, pH levels, and nutrient profile. These effects are highly dependent on the soil properties and CQDs source being used. Prior research indicates that CQDs have the potential to improve soil health, organic C content, and nutrient retention, which can promote plant growth and overall soil health (Vijeata et al. 2022; Sadeghi et al. 2023). For instance, Vijeata et al. (2022) reported that CQDs derived from *Aegle marmelos* did not cause significant changes to soil pH levels which means that their acid neutralizing and base forming effects are tangibly weak. Nonetheless, the application did greatly enhance the oxidizable organic C contents from a low range of 0.100–0.300 and to a doubled medium range of 0.505–0.750, while soil phosphate, nitrate, and ammoniacal N levels were 22–56, 20, and 10 kg ha^−1^, respectively.

In another study, Sadeghi et al. (2023) highlighted that CQDs produced from fungal exopolysaccharides elevated the levels of dissolved organic C content (DOC) by 39.9% in Cd polluted soils and 77.9% in the non-contaminated soils. Moreover, microbial biomass in CQDs treated soils increased by 54.9%, suggesting CQDs ability to stimulate C cycling in soil ecosystem. Additionally, CQDs can act as excellent adsorbents for example, Li et al. (2019) documented that Cd-CQDs can effectively reduce freely dissolved Cd ion concentration in nutrient medium by 22.5% through its adsorption capacity. Similarly, Li et al. (2018a, 2018b) reported that CQDs act as high-performance adsorbents for Cd, with a maximum adsorption capacity of 2.42 mg g^−1^, particularly in soils with a pH range of 4–7.

### Effect of CQDs on soil enzymatic activities

Soil enzymatic activities are excellent mediators of organic matter decomposition and nutrient cycling for sustainable soil health, which makes them sensitive indicators of soil microbial activity. Soil application of nanomaterials like CQDs can modulate the enzymatic activities as well as stimulate or inhibit microbial-mediated biochemical processes in soil (Sahu et al. 2017). Due to their small size and high surface reactivity, CQDs can interact with the soil enzymes either directly or indirectly through impacting soil microbial communities which can influence soil metabolic functions (Tusher 2023). Therefore, understanding CQDs’ potential impacts on soil enzyme dynamics is crucial for understanding the ecological soil systems.

Different soil enzymes including urease and fluorescein diacetate esterase (FDAE) are critical indicators of soil microbial activity due to their fundamental role in nutrient cycling. In a 90-d incubation study, Liu et al. (2015) observed detrimental impacts on soil enzyme activities with the introduction of varying concentrations of carbon-based nanomaterials. This substantial effect was varied based on their dosage levels, exposure time, and the type of enzymes studied. At first, they observed no drastic changes in activity levels of enzymes studied under different treatment levels; however, they observed modest decrease in FDAE activities with increasing exposure duration. Opposite to this, urease exhibited a clear decline in activity with prolonged exposure time and dosage concentration, specifically with higher doses of 10 μg g^−1^. They further demonstrated that this inhibitory effect in urease activity was only temporary as urease activity restored to normal conditions after 30-d of tested incubation. This recovery phase is likely the result of microbial adaptation under these conditions, where microbes might have started to utilize them as an alternative C source. These studies suggest that carbon-based nanomaterials can produce dose-dependent and transitory fluctuations in soil enzyme activities with soil microbes gradually adapting to these conditions.

All above discussed studies highlight the multipurpose role of CQDs as a transformative nano-agent to enhance soil health by stimulating beneficial microbial activity, enhancing nutrient availability and C cycling, and modulating soil enzymatic activities, ultimately leading toward sustainable farming practices. However, long-term field scale studies are required on soil–CQDs interactions to better understand how CQDs would impact soil physio-chemical characteristics and especially soil organic matter stabilization and microbial community resilience. Understanding these interaction dynamics of soil–CQDs is thus fundamental to crafting new strategies ensuring long-term soil vitality and agricultural sustainability.

## CQDs-induced improved tolerance against abiotic stressors

The growth and development of plants relies on certain mineral nutrients and water to be at optimal levels. Nevertheless, during a drought, heavy metal contamination, salinization, extreme temperatures, ultraviolet radiation, and several other factors limit the environment's ability to supersede a minimum budget. These plants have a unique and intricate multidisciplinary and subspecialty response which melds together numerous static and dynamic biochemical, physiological, and molecular processes. Reduced efficiency in photosynthesis is one of the many effects caused by abiotic stress nature. This stems from increased photo-inhibition that occurs concurrently with stomatal closure that restricts CO_2_ use. One of the phenomena which aid in explaining stress-induced growth of plants is the increase in reactive oxygen species (ROS) formation (Younes et al. 2024). These molecules can cause severe damage to vital cellular components like proteins, carbs, fats, and even DNA (Farooq et al. 2019). CQDs and doped CQDs have been reported to mitigate various abiotic stressors, including light, salinity, heat, drought, nutrient deficiencies, and heavy metal toxicity by improving key physiological parameters, such as chlorophyll contents and strengthened antioxidant response that protects the photosynthetic machinery against oxidative damage (Table 5). One significant contributor to enhanced tolerance is the calcium Ca^2+^ signal transduction system, which plays a vital role in plant defense and precedes the release of ROS. CQDs also facilitate stress adaptation by improving Ca^2+^ signaling and acting as ROS scavengers (Li et al. 2020a, b; Li et al. 2023a). Application of CQDs is reported to efficiently relocate Ca^2+^ via CNGCs (cyclic nucleotide-gated channels) while amplifying signaling pathways (Table 5).

**Table 5:** Selected studies showing CQDs-induced improvement (%) in physiological parameters of plants exposed to different abiotic stresses

Sr. no	CQDs type	Plant type	Type of stress	Optimum dose (ppm)	Exposure duration (days)	Improvement (%) in plant physiological parameters	References
1	Mg-CQDs	Rice (*Oryza sativa*)	Salinity	150	23	Chlorophyll (36.2); Carotenoid (16.21); K (21.46) contents	Liu et al. 2024
2	Ce- CQDs	Mung bean (*Vigna radiata*)	Salinity	2	15	Chlorophyll contents (90); POD (77); SOD (76); CAT activity (77); Total protein (76) contents	Dong et al. 2022
3	Mg, N-CQDs	Rice (*Oryza sativa*)	Light	300	16	Chlorophyll a (14.39); Chlorophyll b (26.54); Photosynthetic efficiency (127.16); Electron transport rate (104.48); RuBisCO activity (6.62)	Li et al. 2021
4	Polyacrylic acid-CDs	Maize (*Zea mays*)	Drought	5	-	Net photosynthetic rate (206.8); Root proline contents (36.3); Root ABA contents (6.9)	Wang et al. 2022
5	CQDs & CQDs-Cu	Sunflower (*Helianthus annuus*)	Nutrient deficiency	20	30	Chlorophyll a (24.25); Carotenoid contents (21.33)	Ahmed et al. 2022
6	Biomass-derived carbon dots (B-CDs)	Pakchoi (*Brassica chinensis* L.)	Nutrient deficiency		7	Promoted chlorophyll synthesis (30–100%); Ferredoxin (40–80%); RuBisCO activity (20–110); Net photosynthetic rate (130–300%)	Cheng et al. 2023
7	Fungal exopolysaccharide-derived carbon dots (EPS-CDs)	Maize (*Zea mays*)	Cd^2^⁺	150	10	Chlorophyll content (24.3); Dissolved organic carbon in soil (39.9)	Sadeghi et al. 2023
8	CDs	Chickpea (*Cicer arietinum* L.)	As	3.65	6	Membrane stability (33); Enhanced proline (95%); Glutathione contents (198%)	Chandrakar et al. 2020
9	CQDs	Green mustard (*Brassica juncea*)	–	150	42	CO₂ assimilation rate (58); Rubisco activity (33)	Chowmasundaram et al. 2025

### Ultraviolet radiation and heat stress

Red-emissive carbon dots (RCDs) emit light in the range of 550–800 nm, aligning closely with the red and far-red wavelengths required by plants for efficient photosynthesis. While plants typically absorb UV and yellowish green light less efficiently, RCDs enhance the red and far-red components of sunlight without significantly altering the blue portion of the spectrum, thereby improving overall plant photosynthetic performance (Li et al. 2021). Similarly, Mu and Han, (2024) observed that using magnesium and nitrogen-doped carbon quantum dots (Mg-N-CQDs) to wheat seedlings when exposed to UV-B stress, it can promote both seed germination and development by controlling physiological responses. Sun et al. (2025) reported that Ce-CQDs enhanced wheat seedling tolerance under UV-B stress by increasing the activities of key antioxidant enzymes to detoxify ROS. Godínez-Mendoza et al. (2023) showed that the biomass-based CQDs synthesized from agave bagasse exhibited strong UV-blue luminescent features and enhanced plant growth while shielding against harmful UV rays. These CQDs were also reported to enhance seed germination and development of seedlings. Besides, Wang et al. (2021a, b) showed that applying CQDs to the foliage of Italian lettuce enhanced the activity of POD, SOD, CAT, protein contents, and soluble sugars, while decreasing proline and MDA, which are both signs of osmotic and oxidative stress. By reducing oxidative damage and stabilizing physiological processes, CQDs contribute significantly to plant growth and development under heat stress.

### Salinity

The challenge of salinity is one of the primary aspects affecting agricultural production globally. Plants under salt stress suffer various physiological and biochemical breakdowns, such as ionic and osmotic imbalance, oxidative stress, and stunted growth. The pristine and doped CQDs application has reasonably good salinity mitigation results. Sarkar et al. (2022) investigated the effects of two sugar-terminated carbon nanodots (CNDs) to alleviate salinity stress in *V. radiata* seedlings. Both types of CNDs improved seed germination, chlorophyll pigment components, antioxidant defense mechanisms, and helped increase osmotic and ionic homeostasis. Moreover, it has been shown that CQDs increase the non-enzymatic and enzymatic salt stress antioxidant capabilities of plants. Under conditions of stress, proline is a compound that helps to protect plants from over-damage; however, CQDs aid in suppressing proline accumulation while maintaining ionic balance under salt stress. Abdel Latef et al. (2017) showed that grapevine plants grown under salt stress and treated with CQDs demonstrated functional modulation of proline contents and enhanced salt stress resistance. Likewise, Gohari et al. (2021a) showed that putrescine functionalized CQDs (put-CQDs) alleviated salt stress in grapevine by increasing the fresh and dry weights of leaves, K^+^ content, photosynthetic pigments, chlorophyll fluorescence, proline content, phenolics, and antioxidant enzymatic activities while lowering Na^+^ content, electrolyte leakage (EL), malondialdehyde (MDA), and H_2_O_2_ level. Furthermore, Gohari et al. (2021b) mentioned that proline functionalized CQDs (pro-CQDs) enhanced grapevine tolerance to salinity stress by improving its physiological performance. In addition, Liu et al. (2022) reported that the application of cerium oxide nanoparticles (CeO2 NMs) on the plant leaves enhanced salt tolerance in maize. In addition, Guo et al. (2022a, b) has shown that magnesium–nitrogen co-doped CQDs have positively influenced plant growth and decreased oxidative damage in salinized tobacco plants, suggesting the role that doped CQDs can have in stress alleviation.

Dong et al. (2022) showed further that mung bean seeds treated with Ce-CQDs under saline stress conditions had better root length, leaf length, and height than untreated plants. Moreover, Ce-CQDs improved the functions of the key antioxidant enzymes and decreased the markers of stress, such as MDA and proline. Surprisingly, mung bean plant uptake of Ce-CQDs was associated with a reduction in NaCl uptake of 21.8%; in addition, there was an increase in stomatal opening which led to better metabolic and salt excretion efficiency. Li et al. (2021) showed that CQDs very efficiently non-specifically scavenge ROS in plant cells which prevents oxidative damage and improves salt stress tolerance in lettuce. Considering the promising results, it is important to study biomass-derived CQDs further, so this could lead to improvements in plant tolerance to salinity. Their biodegradability, low toxicity, and sustainable synthesis render CQDs useful for application aimed at improving stress tolerance and crop productivity in agriculture.

### Drought

Environmental concerns, such as water shortages, meteorological droughts, as well as global warming, have considerably worsened in the last twenty years, being referred to as global issues (Zhou 2021). It is expected that during the year 2030, over 20% of developing countries will face water shortages (Cheng et al. 2023). Likewise, agronomical drought has developed as a principal restriction on agricultural output, lowering yields by up to 50% (Kurunc et al. 2021). Thus, enhancing drought resistance in crops is critical for ensuring global food security. In this context, CQDs and other carbon-based NMs have shown promise in mitigating drought stress through nano-agricultural strategies. Under drought stress, CQDs application either via seed priming or foliar application enhances photosynthetic efficiency, improves physiological performance, and ultimately supports crop growth and yield (Wang et al. 2019). Their high electron transfer capability enables CQDs to mitigate drought-induced stress and improve plant growth by enhancing antioxidative defense, water retention, and metabolic activity (Farhangi‑Abriz et al. 2024). In their recent study, Farhangi‑Abriz et al. (2024) reported that foliar application of CQDs in drought-stressed soybean significantly improved numerous agronomic and physiological traits: leaf area (by 21%), leaf water contents, antioxidative activities, osmolyte production, maximum efficiency of photosystem II (by 19%), chlorophyll contents (by 18%), relative photosynthetic electron transport rate (by 3%), ground green cover (by 14%), and grain yield by 25%. Moreover, CQDs decreased ROS production, lipid peroxidation, and the osmotic stress in field grown soybean plants.

Mechanistically, foliar application of CQDs not only improves photosynthesis but also induces the synthesis of signaling molecules such as abscisic acid (ABA) and proline (Pro) in plant leaves. These signals are then transported from leaves to roots, where increased ABA and Pro levels up-regulate aquaporins (AQP) expression, reducing osmotic pressure and improving water uptake. Once drought stress subsides, root-to-shoot signaling downregulates ABA synthesis, promotes stomatal re-opening, and restores normal plant growth (Wang et al. 2022). Yang et al. (2022) further reported that the foliar-applied CQDs improved carbohydrate metabolism and promoted maize growth under the drought stress. Similar findings were reported in wheat by Gong and Dong (2021), who observed improved growth and productivity following foliar application of CQDs.

In addition to foliar application, soil-based delivery of CQDs has also demonstrated great promise. Wang et al. (2022) reported that soil application of CQDs increased the nutritional quality and growth of soybean by regulating rhizosphere processes, improving N bioavailability, and increasing water uptake under drought stress. The surface modification of CQDs with polyacrylic acid increases the chloroplasts'capability of CQD penetration, while the doping of N-CQDs with ROS scavenging functionalities enhances the surface. Wang et al. (2021a, b) noted that foliar application of polyacrylic acid-functionalized N-doped CQDs (PNDs) decreased ROS accumulation, along with improving photosynthetic performance, and increasing root ABA contents.

Proline concentrations enhanced maize growth under drought stress. Additionally, Kou et al. (2021) found that the synthesized NMs from glucose and cysteine via hydrothermal method CQDs, augmented seed water absorption, seed germination rate, and drought resistance index, along with the enhancement of these NMs. Treatment also augmented the activities of POD and SOD, stress-relief dominant antioxidant enzymes. Kara et al. (2023) using molasses-derived carbon dots (MCDs), which enhanced the activities of antioxidative enzymes and proline accumulation while lowering MDA levels, a lipid peroxidation marker, reported a dose-dependent growth enhancement in tobacco plants under drought and salt stresses.

### Nutrient deficiency

Reduced CQDs and doped CQDs can also reduce deficiency in plant nutrients by acting nanocarriers. These NMs with enhanced penetration because of their small size can directly supply required nutrients to nutrient tissues within the plant. When doped with macro- and micronutrients, especially with N, P, and K, these CQDs are capable of augmenting plant growth and productivity. Li et al. (2020a, b) observed the stimulation of growth in rice plants due to the application of CQDs for increasing nutrient supply and uptake into the plants. Li et al. (2023) reported the same for wheat growth because of high permeability of CQDs through biological membranes leading to better nutrient delivery and increased water uptake. Cheng et al. (2023) made CQDs from walnut (*Juglans regia*), soybean (*Glycine max*), walnut *Juglans regia*, and dried laver using hydrothermal method. The released important nutrients like magnesium (2.5%), copper (0.45%), and iron (6.0%) greatly enhanced the growth of lettuce which is otherwise stunted due to nutrient deficiency. Treated plants improved in chlorophyll contents, photosynthesis, biomass accumulation, and normal development. In addition, reports indicated that CQDs enhanced K use efficiency by activating cyclic nucleotide-gated ion channels, which improved root physiology and increased K accumulation. This accumulation further facilitated photosynthesis and mitigated oxidative stress in K-deficient leaves.

### Heavy metal and hydrocarbon

**Fig. 4:**
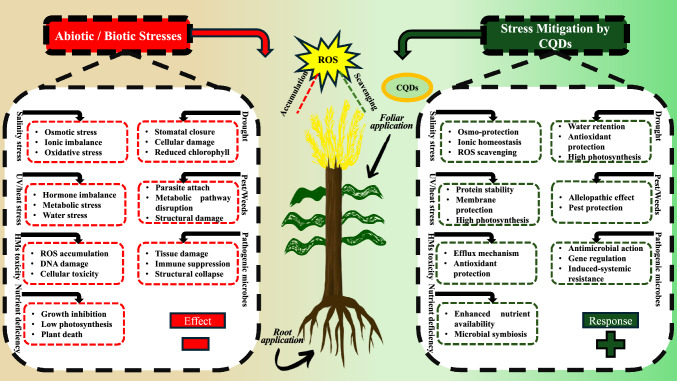
Illustration demonstrating multifaceted roles of CQDs in mitigating plant abiotic (salinity, UV/heat stress, heavy metals toxicity, nutrient deficiency, and drought) and biotic stresses (pests/weeds and pathogenic microbes) through diverse mechanisms

Carbon quantum dots (CQDs) have emerged as a viable option for managing heavy metal stress in plants, chiefly Cd (cadmium) toxicity. As cited by Li et al. (2019), the application of CQDs mitigated the uptake of Cd in Citrus maxima seedlings by over 50% while also decreasing the oxidative stress caused by Cd. This was made possible primarily by the adsorption of Cd ions by CQDs which lowered the translocation of Cd from roots to shoots due to lower free ionic concentration of Cd. Moreover, CQDs enhanced the activities of the antioxidant enzymes which helped in alleviating the Cd damage to plant cellular membranes. Other investigators also monitored the effect of fungal driven CQDs on reducing the Cadmium toxicity in the plants. Sadeghi et al. (2023) showed less Cd uptake, while the chlorophyll contents and dissolved organic matter (DOC), plant dry biomass, and soil organic carbon increased by 68%. These results indicate the capability of bio-derived CQDs to enhance the cadmium stress tolerant capacity of various plants. Apart from cadmium, CQDs have also been reported to reduce plant toxicity of arsenic (As). For example, Chandrakar et al. (2020) noticed that the application of CDs on *Cicer arietinum L*. (Chickpeas) crops mitigated the detrimental effect of asparagus by decreasing the uptake of as and ROS detoxification. In addition, it further contributed to the overexpression of many defenses-related genes and antioxidants including proline and glutathione. Collectively, these findings suggest that CQDs may have the potential to lessen the impact of heavy metal contamination in soils (Fig. 4).

## CQDs-induced improved tolerance against biotic stresses

One of the most challenging biotic strains to control in crop production is the infection by a phytopathogen (Li et al. 2018a, b; Abdelrhim et al. 2021). CQDs are reported to demonstrate great biotic stress alleviation potential through the improvement of nutrient uptake, hormonal modulation, and stimulating the plant's pathogens’ biological defense mechanisms (Fig. 4) (Servin et al. 2015). In addition, the effects of CQDs on phytohormones [e.g., salicylic acid (SA) and jasmonic acid (JA)], which serve protective functions against biotrophic and necrotrophic pests, have also been shown (Wang et al 2021a, b). As an example, Li et al. (2018a, b) noted that the use of CQDs on rice boosted its defense against the blast disease by provoking overexpression of a thionin (Os06g32600) defense gene. This gene codes for a thionin, which is part of the class of pathogenesis-related (PR) proteins. Further, CQDs have the ability to transform into growth hormone acting CO_2_ analogs which enhance plant growth. CO_2_ was transformed into carbohydrates due to the Calvin cycle of photosynthesis further metabolism of plants (Lahiani et al. 2016). Plant energy during periods of stress may also be preserved in the form of carbohydrates which can be utilized to fuel the Calvin cycle. Due to the ability to penetrate plant cells and their biocompatible nature, CQDs have shown broad-spectrum antimicrobial activities against a wide range of phytopathogenic fungi, oomycetes, and bacteria. Wang et al. (2021a, b) used an eco-friendly synthesis method to derive N-doped carbon quantum dots (N-CQDs) from the roots of *Moringa oleifera*. These N-CQDs were able to inhibit *Phytophthora nicotianae* and *Corynespora cassiicola* by 75.3% and 82.8%, respectively. The alternative result was noted by Kostov et al. (2022). In the year of 2022, it was shown that CQDs inhibited actions of *Phytophthora infestans* and other fungal pathogens, such as *Botrytis cinerea, Alternaria alternata*, and *Fusarium oxysporum*. This reduction in the severity of wilt corresponds to the previously noted enhance stimulation of antioxidative enzyme activities in the plants and the reduction of pathogen-induced oxidative stress (Fujita et al. 2022).

Luo and coworkers (2021) discovered that N-CDs were 1.56 times more effective than pristine CDs (P-CDs) and were able to suppress bacterial wilt severity in tomatoes by 71.19%. This lower wilt severity was ascribed to higher stimulation of the antioxidative enzyme activities in the plants, and thereby lessened the oxidative stress caused by the pathogen, showing that enhanced immunity in these plants was supported by strengthened antioxidative defense system, i.e., increased activities of superoxide dismutase (SOD) and peroxidase (POD). The antimicrobial activity of the CDs, that were obtained from cigarette smoke, was emphasized as an activity of broad-spectrum nature, even to the drug-resistant pathogens. Curious quantum dots also demonstrated the ability to decrease the mycelial growth of *Phytophthora infestans* and sporangia of other pathogens, which was illustrated by the decrease in the optical density of spore suspensions. The work of Wang et al. (2014) analyzed the antifungal activity of CDs on *F. graminearum* and *F. poae*, where they hypothesized that the water uptake inhibition and catalysis of plasmolysis were the two main reasons for their antifungal activity due to reactive oxygen species (ROS) formation and plasmolysis. All these studies indicate that CQDs can serve as a promising ecological substitute for chemical pesticides for pathogen control and protection of plants.

## CQDs role in functional genomics and gene expression modulation under stress

The use of different omics technologies, such as transcriptomics, genomics, metabolomics, and proteomics, is transforming the understanding of mechanisms of plant responses to different nanomaterials at molecular levels. These techniques help to elaborate how these nanomaterials alter gene expressions, metabolic pathways, and protein synthesis processes (Chen et al. 2024).

Different studies related to genomic and transcriptomic techniques have showed that exposure to nanomaterials can up-regulate or down-regulate the gene expressions, such as nutrient uptake, hormonal signaling, and stress responses (Khan et al. 2022). The effects of translational modifications and changes in gene expressions are further assessed by Proteomics (Munir et al. 2023). For example, a study was done at proteome level, and it compared the phytotoxicity of three different metal-based nanoparticles (Al_2_O_3_, ZnO, and Ag) in soybean seedling. It was observed that ZnO and Ag nanoparticles severely affect plant growth and root tissues by giving them oxidative stress. Whereas, Al_2_O_3_ had less damage than ZnO and Ag, and it also regulated the proteins involved in responding the oxidative stress and hormonal signaling (Hossain et al. 2016).

Incorporating data from multiple omics technologies into analysis could help us understand the systems better and find markers of nano-toxicity, nutrient uptake by plants, and improved growth. With this knowledge, experts can develop new products for different crops while keeping in mind the safety of the environment (Danai-Varsou et al. 2023). With omics technologies becoming easier to use and accessible, their applications in nano-agriculture are likely to expand to promote sustainability and data-driven innovations along with regulatory goals (Zhang et al. 2021).

### CQDs as nanocarriers for gene delivery in plants

Over recent years, nanotechnology has provided novel ways to plant genetic engineering, with CQDs emerging as viable nanocarriers for gene delivery vehicles. CQDs due to their nanoscale size, excellent biocompatibility, and photoluminescence have been investigated for delivering nucleic acids, such as RNA, DNA, and small interfering RNA (siRNA) into plant cells, allowing for both modulation of gene expression and functional studies. CQDs can be functionalized with numerous chemical groups to improve their capability to bind and transport the genetic material across the plant cell walls and membranes. For instance, carbon-based nanocarriers derived from glucose or saccharose and specified with branched polyethyleneimines (PEI) have been shown to efficiently delivery the double-stranded RNA (dsRNA) into the cucumber leaves, which resulted in a 50-fold rise of dsRNA uptake as compared to the naked dsRNA. This delivery method also resulted in the production of small interfering RNA (siRNA) by 13-fold, demonstrating efficient gene silencing mechanisms (Delgado-Martín et al. 2022). This biomolecule carrying ability of CQDs opens new opportunities for crop development, functional genomics, and stress response studies in plants.

### CQDs applications in gene expression modulation and gene silencing

Gene silencing and expression modulation are both critical methods in plant biotechnology, allowing careful control of gene expression to increase the desirable features while suppressing detrimental ones. These methods, which include mechanisms such as microRNA (miRNA) and RNA interference (RNAi) pathways, influence gene expression at both transcriptional and post-transcriptional levels (Zhao et al. 2024). The use of these methods has considerably expanded crop improvement techniques, enabling improved plant nutritional profiles, better tolerance to environmental challenges, and disease resistant crop varieties (Zhao et al. 2024). The integration of nanotechnology, particularly the use of CQDs, has further augmented these methods by providing effective delivery routes for genetic materials, thereby expanding the potential for precise gene regulation in plants. The successful use of CQDs for gene delivery has been confirmed in several plant species including tomato (*Solanum lycopersicum*) and *Nicotiana benthamiana* (Schwartz et al. 2020), mung bean (*Phaseolus radiatus*), rice (*Oryza sativa japonica*), wheat (*Triticum aestivum*) (Wang et al. 2020), and *Arabidopsis thaliana* (Lin et al. (2023).

Lin et al. (2023) achieved efficient gene delivery and expression regulation in *Arabidopsis thaliana* by demonstrating the CQDs multifunctional role. Their findings showed that CQDs could successfully deliver the plasmid DNA into plant cells by forming stable nanocomplexes with plasmid DNA, which allowed the expression of an Argonaute family genes without the requirement of any mechanical transformation techniques. Their work also demonstrated the intracellular localization and real-time imaging capabilities of CQDs, hence bolstering their application as effective nanocarriers for cellular tracking and gene delivery.

Polyethyleneimine (PEI) and CQDs were combined by Wang et al. (2020b) to develop a nanocomposite (CDP) which served as an effective vector for gene transfection in both plant and animal cells. Their work showed that the expression of functional genes including β-glucuronidase (GUS) and hygromycin resistance gene could be successfully delivered and expressed transiently in a variety of plant tissues, such as wheat leaves, mung bean leaves and roots, and leaves of rice plants. Notably, the CDP offered highly effective DNA delivery system for immediate gene expression in plants by facilitating plasmid transport into cells and shielding DNA from DNase degradation. Schwartz et al. (2020) used low-pressure spray application of CQDs–siRNA formulations in tomato (*Solanum lycopersicum*) and benth (*Nicotiana benthamiana*)*,* which significantly silenced the GFP transgenes and endogenous genes involved in chlorophyll synthesis, including magnesium chelatase subunits. Overall, their study demonstrated the CQDs potential in supporting RNA interference (RNAi) for crop improvement and plant functional genomics.

### Implications under stress conditions

Under conditions of biotic and abiotic stresses, the ability to rapidly modulate gene expression is critical for adaptation and survival of plants. CQDs-mediated delivery methods were recently investigated for their ability to activate plant immunity against viruses, signifying their utility in stress reduction measures. For example, Xu et al. (2023) evaluated the anti-viral efficacy of three dsRNA-loaded NPs-CQDs, amine-functionalized silica nano-powder (ASNP), and chitosan quaternary ammonium salt (CQAS)—against the potato virus Y (PVY) by spraying, root soaking, and infiltration delivery methods. Among these, CQD–dsRNA NPs revealed remarkable efficiency, providing systemic protection for up to 21-d post-application against PYY by foliar spraying. Furthermore, fluorescence imaging confirmed the uptake and transport of these NPs within plant tissues, highlighting the potential of CQDs as effective nanocarriers for dsRNA delivery in plant anti-viral strategies. This efficient delivery of CQD–dsRNA enhanced the RNAi mechanisms to combat viral infections.

In another study, Wang et al. (2020) evaluated the effectiveness of three different NPs-chitosan, Lipofectamine2000, and CQDs—as carriers for dsRNA targeting the glyceraldehyde-3-phosphate dehydrogenase (G3PDH) gene in the rice striped stem borer (*Chilo suppressalis*). Among these, CQD–dsRNA complexes exhibited the highest gene silencing efficiency and larval mortality rates. Their work also demonstrated a strong correlation between RNAi efficiency and hemolymph dsRNA content, indicating the stability and superior delivery of CQD–dsRNA complexes.

Kostov et al. (2022) examined the CQDs dual role in controlling various plant pathogens and improving gene silencing methods. Their findings demonstrated that CQDs not only inhibited the development of oomycete plant pathogens (*Phytophthora infestans)* and other fungal plant pathogens (*Alternaria alternate, Fusarium oxysporum,* and *Botrytis cinerea*) but also enhanced the efficiency of dsRNA-induced gene silencing. The CQDs–dsRNA combination resulted in significant drop in target gene transcripts in developing sporangia, demonstrating CQDs potential as effective nanocarriers for RNAi-based plant protection approaches. Notably, they observed no cytotoxic effects on human keratinocytes at pathogen-effective concentrations, implying their safety for broader agricultural uses. These findings highlight CQDs’ potential as excellent nanocarrier for RNAi-based pest management techniques making them a significant tool for functional research in plant biology and with applications in precision agriculture.

While the current research on CQDs-mediated plant gene delivery illustrates its promising role, further research is needed to optimize the CQDs delivery efficiency, stability in gene modifications, as well as comprehension of its long-term effects. Advancements in CQDs synthesis and functionalization are projected to broaden its uses in plant biotechnology, aiding promote crop improvement strategies and sustainable agriculture.

## CQDs’ applications in agrochemical residue detection

To meet the demands of growing world population, the reliance on chemical fertilizers and various agrochemicals, such as pesticides, herbicides, insecticides, and fungicides, continue to rise (Li et al. 2016). This increasing usage leads to the accumulation of toxic, carcinogenic, and non-biodegradable substances in soil and food ecosystems, posing serious threats to sustainable agriculture and environmental health. Conventional techniques for agrochemical residue detection, including high-performance liquid chromatography (HPLC), gas chromatography, mass spectrometry, enzyme-linked immune-sorbent assays (ELISA), and electrochemical methods, often suffer from limitations, such as high costs, complex sample preparation, and time-consuming procedures. CQDs offer a versatile, cost-effective, and efficient alternative for agrochemical detection (Fig. 4). Their fluorescence quenching or enhancement properties are central to their function as nano-sensors. CQDs-based sensing relies on their interaction with target analytes, primarily through fluorescence quenching mechanisms, such as fluorescence resonance energy transfer (FRET), the inner filter effect (IFE), and photo-induced electron transfer (PET). These mechanisms enable sensitive and selective detection of trace agrochemicals residues. Furthermore, the selectivity and sensitivity of these sensors can be fine-tuned by adjusting CQDs size, surface functionality, and doping of CQDs, enhancing their applicability in various environmental contexts. Some of these sensors developed are CQD-enzyme biosensors, CQD-aptamer APT biosensors, CQD-molecular imprinted polymer MIP sensors, and independent CQD fluorescence biosensors.

### Pesticides and herbicides

CQDs have become some of the most sensitive and selective fluorescent sensors for detecting pestilent compounds. For instance, Joji et al. (2024) reported their detection of numerous pesticides, including tetradifon, hurdane, atrazine, imidacloprid, and diethyl chlorothiophosphate with low detection limits using quenching enabled photoluminescent CQDs. Wu et al. (2017) exhibited FRET-based CQDs sensors of exceptional precision and sensitivity for organophosphate detection. Furthermore, sulfur and nitrogen-doped CQDs were also applied for extremely sensitive detection of carbamate pesticides (Li et al. 2016). Panda et al. (2018) demonstrated “light on” photoluminescent CQD systems for detection of flumioxazin and atrazine and detection limits of 3 pM and 0.05 nM for organophosphates and atrazine, respectively. These results emphasize the flexibility and remarkable effectiveness CQDs in detecting herbicides and pesticides. Along with the benefits of modern breeding practices, applying pesticides and herbicides, integrated agriculture chemicals have inflicted profound pollution in biodiversity, soil, and water sources along with the loss of biological species (Khan et al. 2023). The elevated residue amounts of these products pose threats to health of organism (animal and human) of serious concern (Ali et al. 2021).

### Fungicides and insecticides

Fungicides and insecticides are frequently used to control fungal pathogens and insect pests, but their excessive use leads to serious environmental concerns. Various studies have been conducted toward identifying such residues using efficient fluorescence sensors. For example, Yang et al. (2018) generated those CQDs by electrochemical chemiluminescence methods and applied it as sensor for pentachlorophenol (PCP) which is one of the most widespread herbicides having detection limit of 1.3 × 10⁻^12^ g L⁻^1^. Furthermore, CQDs-based biosensors developed with DNA aptamers have achieved high sensitivity detection of insecticide acetamiprid compared to the FRET-based techniques with lower detection limits. Other advancements include the utilization of molecularly imprinted silica-wrapped CQDs for fipronil identification via fluorescence quenching (Ren et al. 2024) along with an electrochemical sensor to identify 1,4-DCP exhibiting improved catalytic performance (Feng et al. 2024). Such CQD-based sensors feature enhanced sensitivity, decreased detection limit, and can be employed for real-time monitoring.

## Emerging applications of CQDs in food technology, and biomedical and environmental remediation

CQDs have attracted considerable interest beyond their traditional uses in the field of agriculture, emerging as multipurpose nanomaterials with applications spanning food industry, environmental remediation, and biomedicals due to their strong fluorescence, excellent biocompatibility, and tunable surface functionalities, electrical, and optical characteristics (Si et al. 2020; Singh et al. 2024) (Table 6). These properties enable smooth integration of CQDs into water purification processes, active food packaging systems, real-time food quality monitoring devices, and advanced medical therapies (Singh et al. 2024).

**Table 6:** CQDs application in various fields, including photocatalysis, active food packaging, food preservation, fluorescence sensing, food analysis, environmental remediation, drug metabolism tracking, cancer diagnosis, and treatment

Sr. no	Applications	Description	References
1	Photocatalysis	Enhancing photocatalytic effects	Si et al. 2020
2	Active food packaging	Antibacterial and antioxidant properties for active food packaging	Ezati et al. 2022, 2023
3	Food preservation	Antibacterial materials in edible coatings for prolonging shelf life	Fan et al. 2019
4	Fluorescence sensing	Fluorescence sensing platform for detecting Hg^2+^ ions	Zhang and Chen 2014
5	Food analysis	Probes for food analysis, particularly for detecting food safety	Ye et al. 2017
6	Environmental remediation	Detecting heavy metal ions, removing pollutants, and photocatalytic degradation of wastewater contaminants	Si et al. 2020; Rani et al. 2020
7	Drug metabolism tracking	Fluorescent markers for tracking drug metabolism	Badıllı et al. 2020; Tavan et al. 2025
8	Cancer diagnosis and treatment	CQDs-based therapy and delivery systems for cancer diagnosis and treatment	Pareek et al. 2025; Tavan et al. 2025

### Food packaging, safety, and quality monitoring

As functional fillers in active food packaging films, CQDs are excellent candidates due to their remarkable antioxidant and antibacterial properties. In addition, their high-water solubility and biocompatibility enable easy incorporation with various biopolymer films. These films can inhibit the growth of food-borne pathogens. For instance, Ezati et al. (2022) described the incorporation of CQDs in edible food coatings for fresh fruits and vegetables to enhance their shelf life and safety. Fan et al. (2019) demonstrated that applying a chitosan-based coating containing kelp-derived CQDs led to significant improvements in the quality of fresh cucumbers stored at 4 °C by reducing weight loss and suppressing peroxidase activity. Additionally, Zhang and Chen (2014) reported that N-CQDs are effective as a fluorescence sensing platform for the sensitive, label-free detection of Hg^2+^ ions with a detection limit of 0.23 µM. Ye et al. (2017) stated that although CQDs-based probes had great promise in food analysis, their use in food safety monitoring still needs improvement. Recently, the development of intelligent nano-sensors for food quality control has been researched using CQDs (Table 6).

CQDs also have the ability to detect food spoilage. Usually, CQDs react with specific molecules or environmental cues to adjust their optical characteristics. Such variations can be interpreted as changes in food quality/contamination and chemical or physical properties (Ma et al. 2021; Mohammadi and Jafari 2020). Food packaging films embedded with CQDs have the ability to change their color in response to any change in pH due to their fluorescence sensitivity to changes in pH. This gives a visual cue about freshness of food. For instance, fluorescent CQDs synthesized from tangerine peel were used for pH sensing (Ezati et al. 2023).

### Biomedical and environmental applications

CQDs have been used in numerous applications of biomedical field because of their high hydrophilicity, low toxicity, chemical stability, excitation wavelength-dependent photoluminescence, and water solubility (Salvi et al. 2024). For example, CQDs-based drug delivery involves adsorption or binding drugs onto CQDs to achieve site-specific drug delivery with minimum side effects (Lim et al. 2015; Zhu et al. 2015; Guerrero et al. 2021). The bond between drugs and CQDs complex gets broken in acidic environment at the diseased site or under other triggering conditions (Li et al. 2020a, b). This enables smooth delivery of drugs to the targeted sites and free CQDs easily excreted by hepatobiliary or renal system (Tian et al. 2020). CQDs are also used as fluorescent tags to monitor drug metabolism (Badilli et al. 2020). CQDs also serve a substantial role in diagnosis and treatment of cancer-related disorders (Palashuddin et al. 2015; Wang et al. 2015; Zhao and Zhu 2016; Gao et al. 2017; Ghosh et al. 2019). To date, a number of antibacterial and anticancer drugs have already been effectively delivered by several modified CQDs (Molaei 2019; Verma et al. 2020; Mahani et al. 2021).

CQDs have also shown promise in the degradation of environmental pollutants including pharmaceutical toxins such as ciprofloxacin and tetracycline and wastewater treatment (Rasheed et al. 2023; Rani et al. 2020). CQDs large surface area and photoluminescent characteristics allow efficient degradation of these toxins under light irradiation. In their report, Rani et al. (2020) mentioned CQDs’ effectiveness in the detection of heavy metal ions, the removal of organic and inorganic pollutants, and photocatalytic degradation of wastewater.

## Toxicity and environmental impacts of CQDs

The escalating application of carbon quantum dots (CQDs) in solar cells, bioimaging, diodes, electrochromic devices, and chemical sensors raises greater concerns regarding their ecological and biological consequences, especially in agriculture (Omran 2020). The low-cost CQDs are appealing because of their high biocompatibility and photoluminescence, and relatively eco-friendly synthetic pathways. However, with their increasing utilization comes the need to comprehensively evaluate their toxicological profile and governance framework. There is a significant gap between agri-environmental policy and science, because CQDs have not been addressed in preset regulatory frameworks, such as exposure limits (PELs). CQDs have no defined thresholds for limitation or regulation which stands as a policy void in regime of cross science regulation (Liu et al. 2022). CQD toxicity is specifically adverse concerning the synthesis method, particle size, surface charge, dosage, and exposure conditions (Truskewycz et al. 2022). Take, for instance, hydrothermal methods of synthesizing CQDs. These methods tend to produce toxic byproducts such as aldehydes, ketones, and carboxylic acids, which are the result of partial decomposition of organic precursors. These compounds may either generate reactive oxygen species (ROS) or serve as environmental pollutants. Detection is particularly challenging, because the compounds are often non-fluorescent, requiring FTIR, GC–MS, or HPLC for identification (Peralta-Videa et al. 2020).

Functional groups that are covalently bonded to CQDs derive from the reagents used which dictate the method of synthesis, and hence, the toxicity of CQDs. CQDs derived from biomass or green-synthesized pathways are less toxic due to the absence of harsh chemicals. Biologically synthesized CQDs possess no toxic functional groups as opposed to chemically synthesized ones which increases environmental burden. With positive surface charges, smaller CQDs are more likely to be taken up by cells and disrupt membranes (Leal et al. 2023). CQDs have a dose-dependent and species-specific effects in plants. In low doses, they tend to stimulate root and seed germination, while higher doses stifle growth and induce oxidative stress. For instance, Vijeata et al. (2022) observed that 50 ppm of CQDs on *Allium sativum* seedlings did not exhibit any genotoxic effects, however, exposed with more than 100 ppm resulted in chromosomal aberrations, decreased mitotic index which suggested dosage-dependent toxicity. Also, controlled toxicity experiments with microalgae, Scenedesmus obliquus, provided evidence to the theory of dose determination responses (Yao et al. 2018).

In addition to terrestrial systems, CQDs have also exhibited Phyto persistence and bioaccumulation potential in the aquatic system. Li et al. (2020a, b) described a case where 78% of photonic CQDs remained suspended in seawater due to phytotoxic effects resulting from stranded light, which degraded the biomass of the alga *Phaeodactylum tricornutum*. Subsequently, these CQDs were filtered by Artemia salina, which resulted in appetite for feeding and filtration posing significant threats for disproportionate trophic transfer and disruption in the food web of marine ecosystems. In human macrophages and other mammalian cells, the smaller positive CQD morphologies were more cytotoxic. Nevertheless, as Leal et al. (2023) suggest, many CQDs are readily digestible and can be excreted, which may reduce long-term risks. The lack of regulatory exposure limits and longitudinal field data, particularly relating to biosafety in ecological contexts agricultural environments where direct application might be made underscores the need for such research into evaluating chronic exposure, bioaccumulation potential, and ecological risks.

## Conclusions and future perspectives

Carbon quantum dots (CQDs) application has proven useful in multiple disciplines, such as agriculture, environmental monitoring, food safety, and biomedicine. Their small size, high-water solubility, photoluminescence, and surface modification make them stand out as novel nanomaterials. Also, their biocompatibility makes them beneficial in wide array of biological applications, including agriculture both *ex-planta* and *in-planta*. *Ex-planta* benefits include improved soil health characteristics crucial for both plant and human health. Moreover, CQDs in their nutritional context improve plant resilience against chronic crop (a)biotic stresses, ranging from nutritional imbalance, salinity, drought, heat, and heavy metal toxicity as well as pathogens attack. CQDs-induced improved plant growth and productivity is ascribed to enhanced photosynthesis through induced osmotic stress (IOS), which increases antioxidative defense, osmolyte, and hormone secretion downstream. At the same time, CQDs are able to detect pesticides, herbicides, fungicides, and even insecticides via fluorescence which makes them sensitive, safe, and efficient in comparison to traditional methods. Nonetheless, unrestricted use of CQDs poses could raise serious concerns from an environmental or toxicology viewpoint. While low concentrations of CQDs may be helpful, higher concentrations tend to cause oxidative stress and cellular damage, plant mutagenicity, and disrupt balance in aquatic ecosystems. Biologically, CQDs are dependent on the method of synthesis, surface chemistry, concentration, and the conditions to which they are exposed.

The absence of legal frameworks and rigorous long-term assessments of safety outcomes intensifies the scrutiny needed regarding their use. There is an important gap in the literature that deals with the synthesis of environmentally unfriendly byproducts with natural precursors which aims to curtail their toxic byproduct synthesis. There is also an urgent need to develop ecotoxicological studies concerning the behavior, degradation, and accumulation of CQDs in various ecosystems. Agricultural or environmentally useful CQDs will require standardized methodologies and exposure limits for safe use. Moreover, the incorporation of CQDs into smart farming and precision agriculture will transform crop telemetry and inputs management. Designing multifunctional CQDs that construct a sustainable framework on agriculture and environmental safety by integrating precise controlled release with real-time monitoring will greatly advance the field.

## Data Availability

Not applicable.

## Abbreviations

AFM: Atomic force microscopy
CNGCs: Cyclic nucleotide-gated channels
CNMs: Carbon-based nanomaterials
CNTs: Carbon nanotubes
CQDs: Carbon quantum dots
-COOH: Carboxyl
DOC: Dissolved organic carbon content
DMF: Dimethyl formamide
EFB: Empty fruit bunch
EDTA: Ethylenediaminetetraacetic acid
ELISA: Enzyme-linked immune-sorbent assays
FDAE: Fluorescein diacetate esterase
FRET: Fluorescence resonance energy transfer
FTIR: Fourier transform infrared spectroscopy
GQDs: Graphene quantum dots
HPLC: High-performance liquid chromatography
HNO_3_: Nitric acid
H_2_SO_4_: Sulfuric acid
LA: Laser ablation
LecRKs: Lectin receptor kinases
MWCNTs: Multiwalled carbon nanotubes
NaClO_3_: Sodium chlorate
NaHS: Sodium hydrosulfide
NaHSe: Sodium selenide
NMs: Nanomaterials
N-CQDs: Nitrogen-doped CQDs
OH: Hydroxyl
PCP: Pentachlorophenol
P-CDs: Pristine CDs
PET: Photo-induced electron transfer
PD: Polymer dots
PNDs: Polyacrylic acid-functionalized N-doped CQDs
TEM: Transmission electron microscopy
XPS: X-ray photoelectron spectroscopy
XRD: X-ray diffraction
